# A Manual of Examination of the Eyes

**Published:** 1879-05

**Authors:** 


### BIBLIOGRAPHICAL.

A Manual of Examination of the Eyes. A course of lectures delivered at the "Ecole Pratique." By Dr. E. Landolt, Director. Adjoint of the Ophthalmological Laboratory at the Sarbonne, Paris. Translated by Swan M. Burnett, M.D., Lecturer on Opthalmology and Otology in the Medical Department of the University of Georgetown, etc. Revised and enlarged by the author. D. G. Brinton, 115 South Seventh street, Philadelphia. 1879.

This is a volume of three hundred and seven pages that will be found of great benefit to specialists, general practitioners and students. Optical principles of the first importance are treated of in such a comprehensive manner that a complete understanding of their intracacies is readily attainable. The various details of refraction are treated physiologically and pathologically to the fullest extent. The correction of morbid variations is explained and illustrated in a purely scientific method, and yet without obscurity. Examinations by the ophthalmoscope, for acuteness of vision and of the perception of colors, are presented in detail with comments, and the whole supplemented by practical examples. At the end of the volume are two charts, giving in tabulated form the general laws of the movements of the eyes, and of paralysis of muscles of the eyes, producing strabismus, etc.

We unhesitatingly recommend the work, and are satisfied that it will prove inestimable.

